# Medical Obituaries

**Published:** 1855-03

**Authors:** 


### MEDICAL OBITUARIES.

Henry Rodgers, M. D., died recently, of typhoid fever, after a brief illness, at his residence, Newton, Jasper county, Iowa. It is less than a year, since the deceased was a faithful and zealous attendant upon the course of Lectures at the Iowa Medical Department, at the close of which he graduated with honor. We remember him well as a young man of unblemished character, and we knew him as an honorable and judicious physician. A large circle of friends mourn that so worthy a career should so soon be closed; they can ill spare the promise of so much usefulness. But, while we lament this, it is a mournful satisfaction that he has left the example of a worthy character, both as a man and a physician.

Dr. Golding Bird.—Died at Tunbridge Wells, on the 27th of October last, Golding Bird, M. D., F. R. S., aged thirty-nine years. For some months previous to his decease, his failing health had obliged him to relinquish all his professional duties. He had long suffered from an affection of the heart, but the immediate cause of his death arose from kidney disease. He has thus fallen a victim to a malady, the elucidation of which has rendered his name distinguished among the promoters of strict science in Medicine. We need hardly add, he was the well-known writer on *Urinary Deposits*, an admirable work, which opened an almost entirely new field for research to the physician, attained at once a brilliant success, and is still regarded as the highest authority upon the subject. A short time before his death he had an attack of hæmaturia which soon became associated with other and unerring evidence of renal calculus. This was followed by pyelitis, which proved fatal.

PROF. LALLEMAND.—Died recently, at Marseilles, M. Lallemand. He was formerly professor of Clinical Surgery at the University of Montpellier, but was particularly distinguished by his admirable writings on the *Diseases of the Encephalon*, on *Spermatorrhœa*, and last, though not least, his *Clinique Medico-Chirurgicale*.

DR. ROBERT SEMPLE.—Dr. Semple died at his residence, fourteen miles south of Colusa, California, on the 25th of October last, from injuries received by a fall from his horse. He was well known to many of the earlier residents of California, having emigrated from Kentucky, across the plains, in 1845. After having taken a prominent part in the Bear Flag revolution in that year, he located at Benecia. He was a delegate to the convention for the formation of a State Constitution, in August, 1849, and was unanimously chosen President of that body. He, in company with Walter Colton, established the first newspaper in California, at Monterey, in 1846.

GEORGE F. TURNER, M. D.—Died of yellow fever, at Corpus Christi, on the 17th of October, Dr. G. F. Turner, Surgeon of U. S. Army, aged 40 years. The deceased was a distinguished member of the Medical Corps of the U. S. Army. His contributions to medical literature were not numerous. In the first volume (*New Series*) of the New York Journal of Medicine, may be found an exceedingly interesting article from his pen, on *Epidemic Miliary Fever*, as observed at Fort Snelling, Upper Mississippi, in the winter of 1847–'48.

JOHN P. HIESTER, M. D.—Dr. Hiester died in Reading, Pa., on the 15th of September last, aged 51 years. He was born in Berks county, Pa., in 1803. In 1827 he graduated at the University of Pennsylvania, having presented a thesis "*On Indigestion.*" He was a distinguished practitioner, a great lover of botany and chemistry. He was enthusiastically devoted to the great interests of the profession, and was lately President of the Pennsylvania State Medical Society.

SAMUEL PARKMAN, M. D.—Died in Boston, on the 15th of December, Dr. Samuel Parkman. He was a graduate of the Medical Department of Harvard University, in 1838, and for a number of years he was Demonstrator of Anatomy in that Institution. He was also, for some time, one of the surgeons of the Massachusetts General Hospital.

